# Adults who microdose psychedelics report health related motivations and lower levels of anxiety and depression compared to non-microdosers

**DOI:** 10.1038/s41598-021-01811-4

**Published:** 2021-11-18

**Authors:** Joseph M. Rootman, Pamela Kryskow, Kalin Harvey, Paul Stamets, Eesmyal Santos-Brault, Kim P. C. Kuypers, Vince Polito, Francoise Bourzat, Zach Walsh

**Affiliations:** 1 Department of Psychology, University of British Columbia, Kelowna, BC Canada; 2 Department of Family Medicine, University of British Columbia, Vancouver, BC Canada; 3 Quantified Citizen Technologies Inc., Vancouver, BC Canada; 4 Fungi Perfecti, LLC, Olympia, USA; 5 Department of Neuropsychology and Psychopharmacology, Faculty of Psychology and Neuroscience, Maastricht University, Maastricht, The Netherlands; 6 Department of Cognitive Science, Macquarie University, Sydney, Australia; 7 Center for Consciousness Medicine, Redwood City, USA

**Keywords:** Psychology, Human behaviour, Psychiatric disorders, Addiction, ADHD, Anxiety, Autism spectrum disorders, Bipolar disorder, Depression, Obsessive compulsive disorder, Post-traumatic stress disorder, Psychosis, Schizophrenia

## Abstract

The use of psychedelic substances at sub-sensorium ‘*microdoses’,* has gained popular academic interest for reported positive effects on wellness and cognition. The present study describes microdosing practices, motivations and mental health among a sample of self-selected *microdosers* (*n* = 4050) and non-microdosers (*n* = 4653) via a mobile application. Psilocybin was the most commonly used microdose substances in our sample (85%) and we identified diverse microdose practices with regard to dosage, frequency, and the practice of *stacking* which involves combining psilocybin with non-psychedelic substances such as Lion’s Mane mushrooms, chocolate, and niacin. Microdosers were generally similar to non-microdosing controls with regard to demographics, but were more likely to report a history of mental health concerns. Among individuals reporting mental health concerns, microdosers exhibited lower levels of depression, anxiety, and stress across gender. Health and wellness-related motives were the most prominent motives across microdosers in general, and were more prominent among females and among individuals who reported mental health concerns. Our results indicate health and wellness motives and perceived mental health benefits among microdosers, and highlight the need for further research into the mental health consequences of microdosing including studies with rigorous longitudinal designs.

## Introduction

The substances now broadly classified as psychedelics have a very long history of salutary use among Indigenous peoples of the Americas/Turtle Island, including the Mazatec, Huichol, Shipibo, and other nations as well as the pre-Columbian Maya, Olmec, Zapotec, and Aztec societies^1^. These long-standing Indigenous health technologies have been subject to centuries of aggressive suppression, first through colonization and the Inquisition of the Americas and later by the US-led “war on drugs”^2^. Nonetheless, they have reemerged over the past several decades as medicines with the potential to address mental illness and enhance well-being among largely non-Indigenous communities. Although this interest has focused predominantly on doses sufficient to engender dramatic alterations in consciousness, the use of smaller *microdoses,* absent from the profound sensory and cognitive effects that typify the psychedelic experience is also a topic of substantial interest in psychedelic interest groups^3^, popular culture^4,5^ and emerging scientific literature^6^. Indeed, whereas long-standing consumption of larger doses of psilocybin-containing fungi is well-documented among the Mazatec people in Mexico, the use of smaller microdoses to support the healing of physical conditions and emotional states such as sadness, anger, envy, isolation and agitation is also common amongst the Mazatec people^7^.

The exact parameters of what constitutes a microdose and the associated practice of regular microdosing have yet to be firmly articulated; however, microdosing has been generally described to involve successive self-administration within a limited time window, of doses of psychedelics that do not impair normal functioning and are predominantly sub-sensorium^8^. Psilocybin and LSD are the substances used by the vast majority of participants in observational and retrospective research on microdosing^9–15^. Reported microdoses identified in observational research typically range from 5 to 20 μg of LSD and from 0.1 to 0.3 g of dried psilocybin mushrooms^9,12–15^. Microdoses are most commonly used several times a week with various patterns of alternating days^9,10,12–17^. The lone study to compare microdosing frequency across LSD and psilocybin users reported equivalent use patterns across substances but did not examine differences in relative dosage^13^.

In addition to microdosing psychedelics alone, growing interest has focused on a practice referred to colloquially as *stacking* which involves combining microdoses of psychedelics—primarily psilocybin-containing mushrooms—with other substances that are proposed to accentuate salutary effects. The use of such admixtures appear to have a long history; Aztecs combined cacao with psilocybin mushrooms in a practice referred to as "cacahua-xochitl", which literally means "chocolate-mushrooms"^18^, and psilocybin admixtures composed of honey, flowers and herbs have been noted in historical records among both Indigenous and non-indigenous peoples^19,20^. Following a similar profile, mushrooms, henbane (also known as nightshade, *Hyoscyamus niger*), stinging nettles (*Urtica dioica*) and other active substances were commonly added to enhance the effects of beer during the Middle Ages until the passage, in 1516, of the German Reinheitsgebot, also known as the Bavarian Purity Beer Act^21,22^. Chocolate and cacao remain popular additions to psilocybin, whereas as adding Syrian rue (*Peganum harmala*), Lion’s Mane mushrooms (*Hericium erinaceus*) and/or niacin appear to be more recent phenomena^21^.

Similar to the practice of microdosing more broadly, the popularity of *stacking* likely emanates from the proliferation of positive anecdotal reports over the past decade rather than from a strong empirical basis. Indeed, to our knowledge no studies have directly tested potential synergistic effects of these substances when combined with psilocybin. As such, the proposed mechanisms of action, benefits and subjective effects of stacked psilocybin microdose admixtures typically follow from reports associated with the stacked substance in isolation. For example, the benefits of cacao^23^ and potential cognitive enhancing properties of Lion’s Mane mushrooms^24^ have been proposed to synergize with the putatively complementary qualities of psilocybin mushrooms^25,26^. Other rationales for stacking include observations regarding potential biochemical interactions. Specifically, both Syrian Rue and Lion’s Mane have been identified as inhibitors of monoamine oxidase (MAOI)^27,28^, and MAOI have a long history of use in psychedelic admixtures such as ayahuasca where they serve to extend and enhance the effects of 5HT2a receptor agonists^29^. In contrast, the flushing effects of niacin are suggested to facilitate psilocybin bioavailability and be prophylactic for abuse^21^. However, despite traditional practices, theoretical rationale, and contemporary anecdote that suggest potential benefits of stacking, empirical studies of most stacked substances are limited and largely involve animal models. As such, caution is warranted when interpreting claims related to the synergistic effects of stacked substances and psilocybin in humans.

The practice of microdosing appears to have increased substantially over the past decade^3^ and recent studies have begun to characterize individuals who microdose^3,9,15^. Comparisons to non-microdosing community samples generally identify few differences between microdosers and non-microdosers^15^. However, some findings suggest that, like psychedelic users more broadly, microdosers are disproportionately male and lower in education and income relative to non-microdosers^9,30^. Interestingly, microdosers report higher levels of past year substance use but lower levels of substance use disorders, anxiety disorders, and negative emotionality^3,15^.

Surveys identify diverse motivations for microdosing; respondents note reducing anxiety and depression, improving well-being, and enhancing cognitive performance as key motivations; less prominent motivations include improving physical health, and enhancing empathy, spirituality, and curiosity^10,12,13^. The prominence of addressing mental health concerns and enhancing psychological well-being and cognition suggest that a substantial proportion of those who microdose may be doing so in an attempt to treat symptoms of mental illness or prevent cognitive decline. Indeed, microdosers report reduced stress^14^, improvements in mood^3,13,17,31^ and attenuation of symptoms of depression^9,14,17^, anxiety^9,13,14^, post-traumatic stress, and obsessive–compulsive disorder^17^. Studies have also reported that microdosing may be perceived as more effective than conventional treatments for psychiatric symptoms^11,13^. Findings from the lone prospective study of microdosing suggest positive changes in most psychological domains on microdose days relative to baseline days^14^, and cross-sectional findings suggest lower levels of dysfunctional attitudes and negative emotionality and higher levels of positive mood^3^. However, although one study has queried the lifetime prevalence of psychiatric disorders among microdosers^15^, no studies have estimated the extent to which psychological differences between microdosers and non-microdosers vary according to mental health history and motives.

The present study reports baseline data from an ongoing longitudinal study of microdosing (Microdose.me), which, to our knowledge, constitutes the largest study to date of microdosing. We aim to contribute to the literature on microdosing by further characterizing microdosers and microdosing practices, including a detailed assessment of combining psychedelic and non-psychedelic substances (i.e., stacking). We test differences between microdosers and controls on depression, anxiety and stress symptoms among participants with mental health concerns and examine relationships between motivation for microdosing and mental health. Finally, we investigate the consistency of microdosing practices and motivations across gender and mental health.

## Methods

### Design and participants

We collected cross-sectional data between November 2019 and July 2020 from self-selected respondents recruited via media related to psychedelic use such as podcasts and online psychedelic research conference presentations. Participants were directed to the Microdose.me website at https://microdose.me/. The website directed participants to install the Quantified Citizen (QC) application^32^ to their Apple mobile device. The QC application was only available on Apple iOS devices at the time of study; as such, participants were limited to iPhone users. The application hosted the study and participants completed questionnaires and assessments entirely within the application. To encourage participation, users were explicitly not asked to submit any personally identifiable information and use of the application was designed to be completely anonymous. All participants endorsed being 18 years of age or older and capable of responding to an English survey. Nonetheless, given the anonymous nature of the study design, these inclusion criteria could not be verified beyond participant self-report. All participants provided informed consent prior to study initiation. Data are drawn from the baseline and supplementary questionnaires from a longitudinal study of microdosing and mental health and consisted of a maximal total of 123 questions, organized hierarchically such that many items were contingent on prior responses. The questionnaire was developed based on previous research and consultations with experts in the field. The study was approved by the University of British Columbia Behavioural Research Ethics Board (H19-03051) and all methods were carried out in accordance with their guidelines and regulations.

Participants reported demographic data and detailed information on microdosing practices as well as use of psychoactive substances. Participants were classified as *microdosers* or *non-microdoser* based on the response to the question “*Are you currently engaged in a regular practice of microdosing?”*. The microdoser group was restricted to individuals reporting a current microdose practice at the time of study participation. The non-microdose group included those who had never microdosed and those with a history of microdosing who were not microdosing during the study. Microdosers were asked to specify microdosing substance, dosage, stacking practice, timing protocol, quantity, duration since microdosing initiation, and motivations for microdosing. Psilocybin and LSD microdose amount was converted to low, medium, and high doses based on previous observational studies^11–15^; for LSD: Low ≤ 10 μg, Medium = 11–20 μg, High ≥ 21 µg and for psilocybin: Low ≤ 0.1 g dried mushrooms, Medium = 0.1–0.3 g dried mushrooms, High ≥ 0.3 g dried mushrooms. Alcohol use, cannabis use, and nicotine use frequency were also assessed, as were past year and lifetime frequency of large, overtly psychedelic doses.

Mental health was assessed with the questions “*Do you currently have any psychological, mental health or addiction concerns*?” Participants who endorsed concerns identified specific mental health and substance use categories from a drop down menu, and were allowed to select more than one category. Motives were assessed through the question “*Why did you start microdosing”,* 18 response options were provided to participants including one opportunity to enter a free text response. Response options assessing motives were generated based on previous research^11–13^ and consultation with experts in the field. Following completion of the baseline survey, participants were invited to follow a separate link within the app to complete the *Depression, Anxiety, Stress Scale-21* (DASS-21)^33^. The DASS-21 contains three subscales assessing *Depression, Anxiety* and *Stress* each of which has 7-items scored from 0 to 3, to assess symptom severity during the past week. The DASS-21 assessment complemented dichotomous baseline mental health questions.

### Statistical analyses

We used *X*^2^ to compare demographic variables, substance use, and mental health conditions of *microdosers* who reported engaging in a regular practice of microdosing at the time of survey response to *non-microdosers* who did not report a current microdosing practice. Analyses restricted to microdosers used *X*^2^ to compare LSD and psilocybin microdosers on frequency, dose, stacking practice and motivations. Comparisons were restricted to LSD and psilocybin, as these substances constitute the vast majority of microdosing in prior studies^3,9,15^. Comparisons of motivations for microdosing across gender and mental health also used *X*^2^. Adjusted residuals were used to identify statistically significant differences. Due to the large number of comparisons, all *X*^2^ significance testing was conducted at the *p* < 0.01 level in order to control for an inflated type 1 error rate associated with multiple comparisons.

Univariate ANOVA tests were conducted to compare microdosers and non-microdosers on DASS-21 *Depression, Anxiety and Stress* subscale scores. Given that the DASS-21 is intended to measure clinical symptom severity^33^, this analysis was limited to participants that reported a mental health condition. This analysis was supplemented with a 2 × 2 univariate ANOVA with microdose status (Microdosers/Non-microdosers) and gender (Male/Female). Further supplementary analyses were conducted with the sample limited to participants who reported a previous experience with large-dose psychedelics in order to control for potential influence of larger dose psychedelic use on DASS-21 scores.

## Results

### Demographics

**Figure 1:**
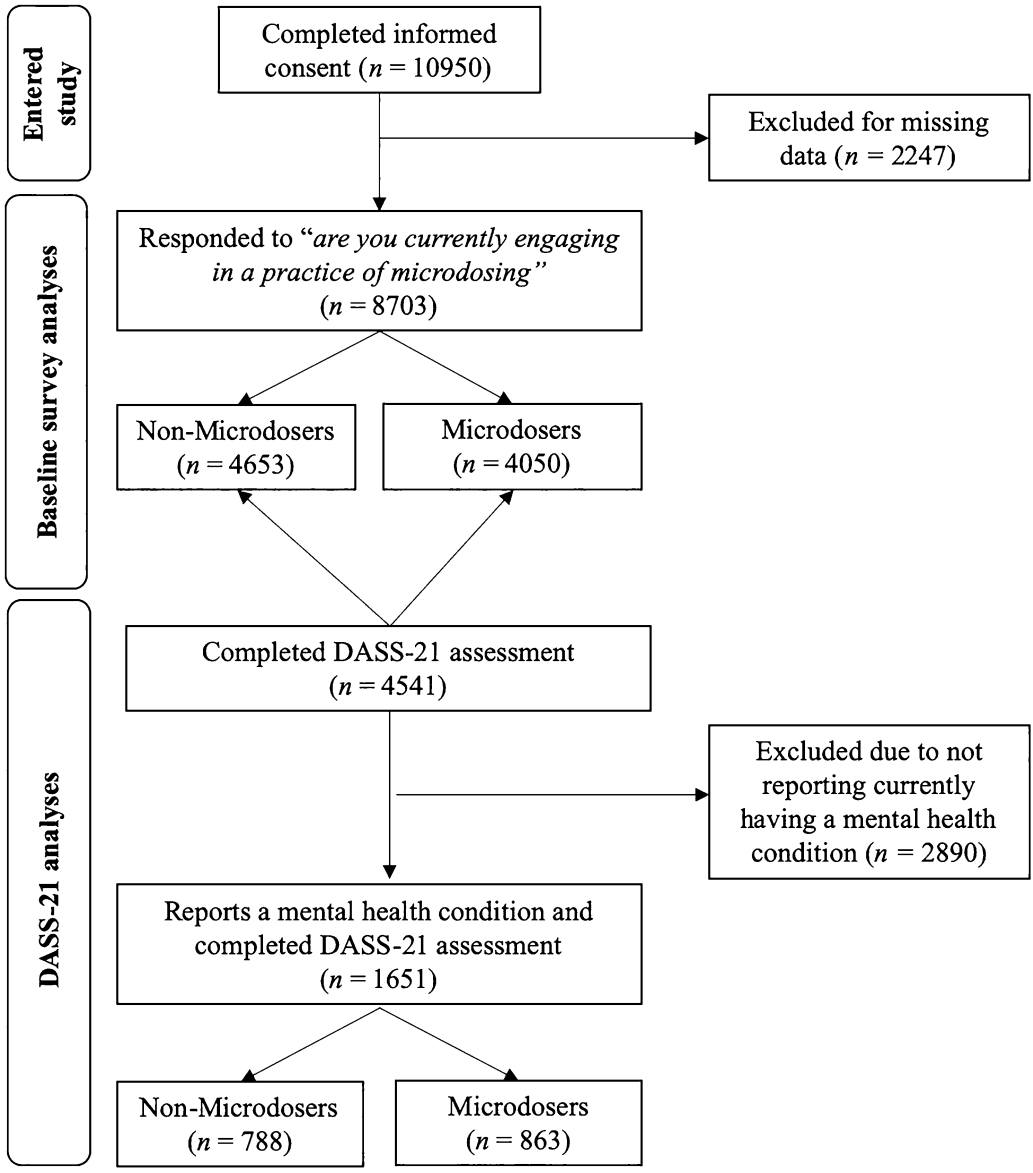
Consort flow chart depicting sample sizes at different levels of analysis.

The baseline survey was completed by 8703 respondents from 84 nations (Fig. 1). Nationalities that compromised more than 1% of respondents were the USA (62.2%, *n* = 5413), Canada (12.7%, *n* = 1104), Australia (4.2%, *n* = 366), Great Britain (4.2%, *n* = 366), Russia (1.4%, *n* = 121), the Netherlands (1.3%, *n* = 111) and Denmark (1%, *n* = 89). Microdosing was reported by 4050 (46.5%) respondents. Compared with non-microdosers (53.5% (*n* = 4653), microdosers were more likely to be older (χ^2^ = 44.91, *p* < 0.01) and to live in an urban rather than a suburban area (χ^2^ = 26.27, *p* < 0.01; Table 1).

**Table 1:** Demographic characteristics.

	Microdosers (*n* = 4050)	Non-microdosers (*n* = 4653*)*
**Gender**		
Male	76.4% (3060)	77.4% (3575)
Female	23.0% (920)	22.0% (1018)
Transgender/non-binary/other	0.6% (25)	0.6% (28)
**Sexual orientation**		
Straight/heterosexual	88.8% (3595)	89.9% (4184)
LGBTQ2S+	11.2% (455)	10.1% (469)
**Age**		
18–24*	19.1% (765)	25.1% (1160)
25–54*	69.9% (2800)	64.4% (2974)
55+	10.9% (438)	10.5% (485)
**Employment**		
Full-time	62.5% (2503)	62.8% (2905)
Part-time	12.5% (502)	12.1% (561)
Student*	9.2% (370)	11.0% (508)
Other	15.8% (633)	14.1% (653)
**Income**		
Under $10,000	6.6% (252)	6.9% (301)
$10,000-$29,999	17.2% (652)	17.1% (749)
$30,000-$89,999	44.7% (1699)	44.3% (1937)
Above $90,000	31.5% (1198)	31.6% (1383)
**Education**		
Graduate degree	13.6% (536)	13.2% (601)
Post-secondary	55.2% (2179)	54.2% (2476)
Secondary	29.2% (1154)	31.1% (1423)
Less than secondary education	2.0% (80)	1.5% (70)
**Community setting**		
Suburban*	39.5% (1558)	44.9% (2047)
Urban*	45% (1776)	40.4% (1842)
Rural	15.5% (612)	14.6% (667)

On variables with missing data, percentages reflect proportions of the total valid, non-missing, responses within a category.

*p < 0.01.

### Mental health and substance use

Mental health or substance use concerns were reported by 29% of respondents, with the most frequently endorsed being anxiety, depression, and post-traumatic stress disorder (PTSD)/trauma-related symptoms, followed by tobacco addiction, problematic cannabis use, problematic alcohol use and panic attacks (Table 2). Less frequently endorsed concerns included bipolar disorder (2%, *n* = 161), eating disorder (2%, *n* = 181), opioid addiction (1%, *n* = 74) and schizophrenia (< 1%, *n* = 18). The sample evinced relatively high levels of substance use with 78% (*n* = 6760) reporting past year cannabis use.

**Table 2:** Mental health and substance use.

	Microdosers (*n = *4050)	Non-microdosers (*n = *4653)	All (*n = *8703)
**Endorsed mental health and substance use problems**			
Any**	32% (1261)	27% (1222)	29% (2483)
Anxiety	22% (878)	18% (810)	20% (1688)
Depression**	21% (842)	17% (754)	19% (1596)
PTSD/trauma-related**	10% (405)	7% (328)	9% (733)
Tobacco dependence*	6% (252)	6% (284)	6% (536)
Cannabis dependence	6% (246)	5% (247)	6% (493)
Alcohol dependence	4% (170)	3% (152)	4% (322)
Opioid dependence	3.5% (44)	2.5% (30)	3% (74)
Gambling dependence	0.7% (9)	0.9% (11)	0.8% (20)
Panic attacks	4% (164)	3% (149)	4% (313)
Schizophrenia	0.7% (9)	0.7% (9)	0.7% (18)
Bipolar disorder	6.4% (81)	6.5% (80)	6.5% (161)
Eating disorder	7.3% (92)	7.3% (89)	7.3% (181)
Learning disorder	5.5% (69)	5.7% (70)	5.6% (139)
Autism spectrum disorder	2% (25)	2.1% (26)	2.1% (51)
**Alcohol use frequency**			
> 3× week**	18% (716)	20% (950)	19% (1666)
≤ 2× week**	62% (2514)	65% (3023)	64% (5537)
Never**	20% (820)	15% (678)	17% (1498)
**Cannabis use frequency**			
> 3× week**	43% (1749)	40% (1858)	41% (3607)
≤ 2× week*	35% (1422)	37% (1731)	36% (3153)
Never	22% (879)	23% (1063)	22% (1942)
**Nicotine use frequency**			
Once per day or more	20% (819)	20% (946)	20% (1765)
Never*	60% (2411)	57% (2666)	58% (5077)

On variables with missing data, percentages reflect proportions of the total valid, non-missing, responses within a category.

*p < 0.05, **p < 0.01.

Microdosers were more likely to endorse any mental health or substance use concern (χ^2^ = 26.89, *p* < 0.01), and were also specifically more likely to endorse depression (χ^2^ = 6.95, *p* < 0.01), PTSD / trauma (χ^2^ = 8.92, *p* < 0.01), and tobacco dependence (χ^2^ = 3.88, *p* < 0.05) but did not differ from non-microdosers with regard to anxiety (χ^2^ = 3.19, *p* = 0.07), problematic cannabis use (χ^2^ = 2.54, *p* = 0.11), problematic alcohol use (χ^2^ = .598, *p* = 0.44), problematic opioid use (χ^2^ = 2.30, *p* = 0.13), problematic gambling (χ^2^ = 0.27, *p* = 0.60), panic attacks (χ^2^ = 0.37, *p* = 0.54), schizophrenia (χ^2^ < 0.01, *p* = 0.95), bipolar disorder (χ^2^ = 0.02, *p* = 0.90), eating disorder (χ^2^ < 0.01, *p* = 0.99), learning disabilities (χ^2^ = 0.08, *p* = 0.78), or autism spectrum disorder (χ^2^ = 0.06, *p* = 0.80). With regard to substance use frequencies, microdosers were less likely to use alcohol frequently and were more likely to abstain from alcohol entirely. Microdosers were also more likely to abstain from tobacco and more likely to use cannabis more frequently (Table 2).

**Figure 2:**
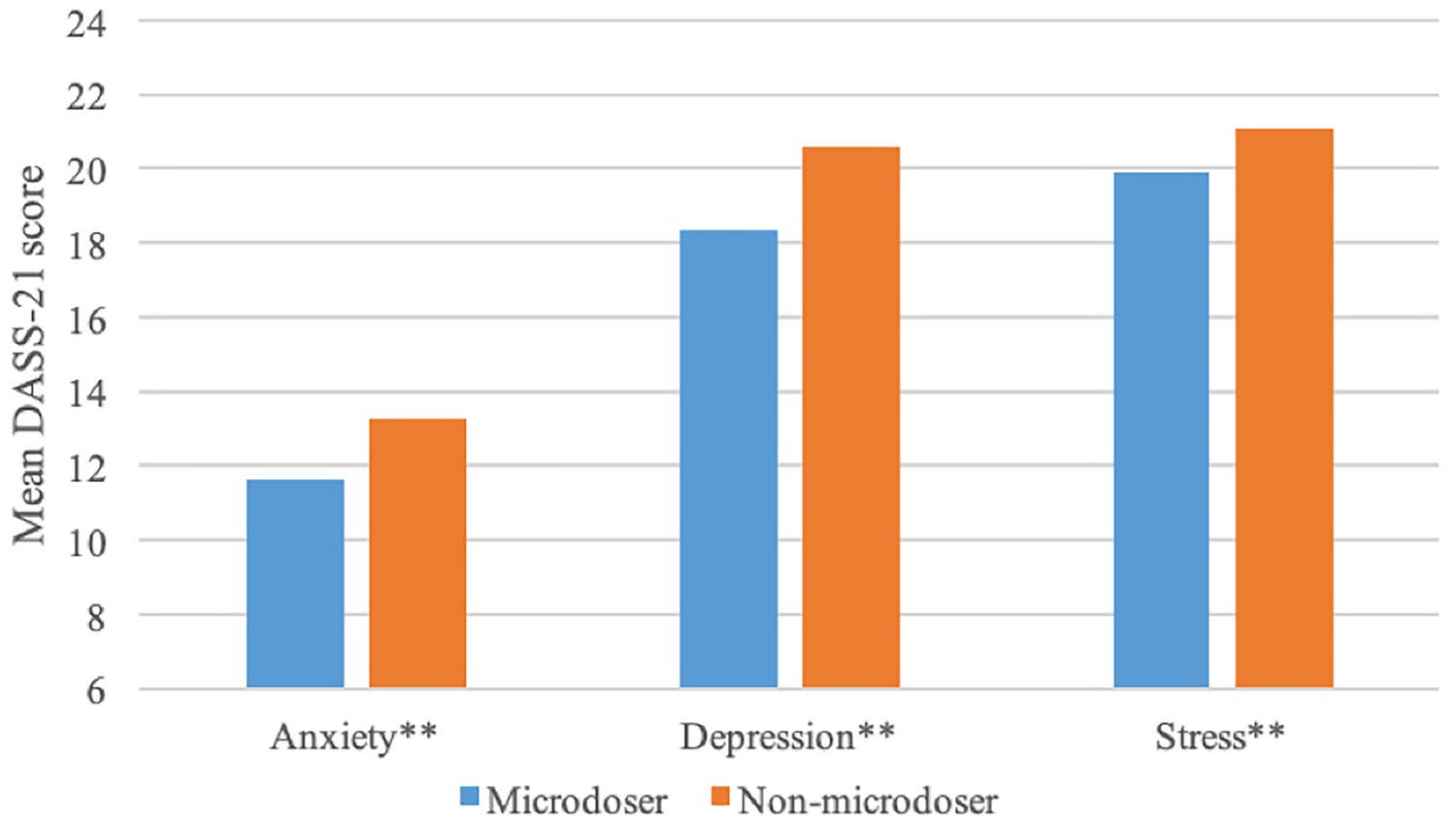
Microdosing and mental health. *Note:* **p < 0.01, *n* = 1651, anxiety, depression and stress drawn from DASS-21 subscales (range: 0–42).

Among the subset of respondents who endorsed currently having a mental health or addiction concerns and who completed the *Depression, Anxiety,* and *Stress* subscales of the DASS-21 (Fig. 1), microdosers (*n* = 863) demonstrated lower scores than non-microdosers (*n* = 788) on *Anxiety* (M = 11.64 (8.98) *vs*. M = 13.22 (9.32); *F* (1, 1649) = 12.26, *p* < 0.01, *d* = 0.17), *Depression* (M = 18.34 (11.96) *vs.* M = 20.58 (11.70); *F* (1, 1649) = 14.71, *p* < 0.01, *d* = 0.19), and *Stress* (M = 19.90 (10.44) *vs*. M = 21.10 (9.40); *F* (1, 1649) = 6.00, *p* = 0.01, *d* = 0.12) (Fig. 2). Gender analyses indicated relative equivalence across males and females (for gender X microdose interactions all *Fs* (3, 1616) < 2.00, *p*s > 0.15).

Most respondents reported lifetime use of larger doses of psilocybin or LSD (87%, n = 7561), and respondents who microdosed reported higher levels of lifetime use than did non-microdosers (92%, (*n* = 3718) *vs.* 83% (*n* = 3843); χ^2^ = 161.13, *p* < 0.01). In light of evidence for the potentially long lasting salutary effects of large doses psychedelics such as psilocybin for depression^34^ we conducted a set of supplementary analyses of differences in *depression, anxiety* and *stress* that was restricted to the subset of respondents who endorsed lifetime use of larger psychedelic doses of psilocybin or LSD. These analyses revealed an equivalent pattern of results to the primary analyses in that microdosers demonstrated lower levels of *Depression, Anxiety* and *Stress* relative to non-microdosing participants (all values for *F* (1, 1433) > 6.00, *p* < 0.01).

### Motives

The most widely endorsed motivation for microdosing was *Enhancing Mindfulness,* followed by *Improving Mood*, *Enhancing Creativity* and *Enhancing Learning* (Table 3). Respondents with and without mental health and substance use concerns differed in proportion endorsing all motives except for *Enhancing Mindfulness* and *Enhancing Creativity*, which were highly endorsed across groups. Respondents without mental health concerns were more likely to report microdosing to *Enhance Learning* (χ^2^ = 10.42, *p* < 0.01), whereas respondents who reported mental health concerns were more motivated to *Reduce Anxiety* (χ^2^ = 336.97, *p* < 0.01)*, Decrease Substance Use* (χ^2^ = 239.27, *p* < 0.01)*,* and *Improve Mood* (χ^2^ = 130.69, *p* < 0.01; Table 3). Female respondents were more likely to report microdosing to *Improve Mood* (χ^2^ = 7.28, *p* < 0.01)*,* and *Decrease Anxiety* (χ^2^ = 55.45, *p* < 0.01)*,* whereas males were more likely to endorse *Enhancing Learning* (χ^2^ = 31.11, *p* < 0.01)*, Increasing Sociability* (χ^2^ = 16.05, *p* < 0.01)*,* and *Decreasing Substance Use* (χ^2^ = 14.41, *p* < 0.01)*.*

**Table 3:** Microdosing motives of respondents with and without mental health concerns.

	No mental health concerns (*n* = 2665)	Has mental health concerns (*n* = 1261)	All (*n* = 4050)
**Motivation**			
Enhance mindfulness	82.0% (2184)	84.9% (1070)	82.9% (3356)
Improve mood**	70.6% (1882)	87.3% (1104)	76.1% (3083)
Enhance creativity	75.3% (2006)	72.2% (911)	74.1% (3000)
Enhance learning**	60.0% (1599)	54.6% (688)	58.1% (2353)
Decrease anxiety**	47.0% (1252)	78.0% (984)	57.4% (2325)
Improve health habits**	41.9% (1116)	53.0% (668)	45.6% (1846)
Decrease procrastination**	40.4% (1077)	53.1% (669)	44.6% (1807)
Increase sociability**	39.9% (1063)	51.3% (647)	43.6% (1767)
Improve sleep**	25.4% (678)	33.1% (418)	28.2% (1141)
Decrease substance use**	18.3% (489)	41.5% (523)	25.8% (1046)

**p < 0.01.

### Microdosing practices

Psilocybin was most highly endorsed as the primary microdosing substance; over 85% of respondents reporting use of psilocybin compared to approximately 11% reporting LSD (Table 4). Notable differences between psilocybin and LSD microdosers included greater likelihood of using higher or medium-sized microdoses among the psilocybin group (χ^2^ = 326.48; *for High Dose*, *p* < 0.01; *Medium Dose p* < 0.01). The modal rate of use was 1–4 times per week for both groups, however the psilocybin group demonstrated greater likelihood of daily or near-daily use (χ^2^ = 77.76, *p* < 0.01).

**Table 4:** Microdosing practices.

	LSD (*n* = 447)	Psilocybin (*n* = 3486)	All (*n* = 3933)
**Dose**			
High**	7.6% (34)	12.5% (435)	11.9% (469)
Medium**	40.1% (179)	71.6% (2497)	30.7% (2676)
Low**	52.2% (233)	15.9% (352)	20.0% (787)
**Quantity**			
5 or more times per week**	6.5% (29)	23.0% (800)	21.4% (866)
1–4 times per week**	83.9% (375)	72.4% (2520)	73.1% (2959)
Combination/ stacking*	26.0% (115)	54.7% (1890)	51.1% (2049)
**Motivation**			
Enhance mindfulness	84.1% (376)	82.8% (2888)	82.9% (3356)
Improve mood	76.3% (341)	76.1% (2652)	76.1% (3083)
Enhance creativity	76.1% (340)	74.0% (2580)	74.1% (3000)
Enhance learning	57.7% (258)	58.5% (2038)	58.1% (2353)
Decrease anxiety**	46.1% (206)	58.9% (2052)	57.4% (2325)
Improve health habits	44.7% (200)	45.6% (1589)	45.6% (1846)
Decrease procrastination	46.3% (207)	44.4% (1549)	44.6% (1807)
Increase sociability	46.1% (206)	43.1% (1503)	43.6% (1767)
Improve sleep**	21.5% (96)	28.8% (1003)	28.2% (1141)
Decrease substance use	21.9% (98)	26.2% (912)	25.8% (1046)

On variables with missing data, percentages reflect proportions of the total valid, non-missing, responses within a category.

*p < 0.05, **p < 0.01.

Users of psilocybin and LSD micorodoses also evinced some differences with regard to motivation for microdosing such that psilocybin microdosers were more likely to endorse *Decreasing Anxiety* (χ^2^ = 26.46, *p* < 0.01) and *Improving Sleep* (χ^2^ = 10.47, *p* < 0.01) as motives. Comparisons of psilocybin to LSD microdosers indicated no differences on measures of *Depression, Anxiety* and *Stress* from the DASS-21 (all values for *F* (1, 827) < 1.0, *p* > 0.50).

Psilocybin users were more likely than LSD users to combine psilocybin with other substances in the process referred to as stacking (χ^2^ = 29.37, *p* < 0.01). The ranking of the most popular type of stacking was the same for psilocybin and LSD, with addition of Lion’s Mane mushroom reported by 39% of microdosers (*n* = 1575), niacin (18%, *n* = 727), and chocolate (5%, *n* = 217). Additionally, 16% of microdosers reported the combination of Lion’s Mane and niacin (*n* = 662). Comparison of microdosers who stacked to those who did not with regard to *Depression, Anxi*ety and *Stress* identified no differences (all values for *F* (1, 849) < 3.0, *p* > 0.10). With regard to motivations for microdosing, respondents who reported stacking were more likely to endorse *Enhancing Creativity* (77% *vs*. 71%, χ^2^ = 19.30, *p* < 0.01) and *Enhancing Learning* (56% *vs*. 44%, χ^2^ = 54.62, *p* < 0.01).

## Discussion

Our characterization of individuals who microdose is generally consistent with those of other cross-sectional studies of microdosing in that psilocybin and LSD were identified as the most frequently used microdosing substances, and the majority of participants reported microdosing between 1 and 4 times per week^3,9,15^. Our findings are also congruent with other studies that have identified prominent microdosing motives of enhancing emotional well-being and cognitive functioning^10–13^. The present results add to prior research that has identified positive associations between microdosing and mental health, and are the first to report associations between microdosing and reduced severity of symptoms of depression, anxiety and stress among adults with reported mental health concerns. Our sample evinced interesting differences with prior research in the relative prominence of psilocybin and LSD; whereas prior observational studies of microdosing reported that LSD was more widely used^3,9,15^, our sample reported much higher rates of psilocybin use. Finally, our novel investigations into “stacking” practices revealed that more than half of the microdose sample combines their microdose substance with another substance such as Lion’s Mane mushrooms or chocolate.

The inclusion of a non-microdose comparison group that was similar with regard to important demographics, substance use, and mental health factors constitutes a distinct contribution of our findings to understanding mental health and microdosing. Specifically, approximately one third of our respondents reported concerns related to mental health and substance use, and, among participants who reported such concerns, microdosing was associated with reduced depression, anxiety and stress symptom severity. In addition, participants who reported mental health concerns were also more likely to report mental health-related motives for microdosing, whereas those who did not report mental health concerns were more likely to endorse motives related to enhancing learning and creativity. Taken together, this pattern of associations suggests that a considerable proportion of those who microdose do so with therapeutic intent to treat mental health symptoms and conditions, and that those who do so appear to be slightly less symptomatic of depression and anxiety than their peers who report similar mental health concerns but do not microdose. Carefully controlled clinical trials are required to more confidently elucidate the potential risks and benefits of psychedelic microdosing, however, the present findings suggest that microdosing psychedelics does not appear to be associated with increased acute negative outcomes, even among potentially vulnerable groups such as those with mental health concerns. Although the cross-sectional design of this study precludes causal inference, these findings from a large international sample of microdosers and a similar non-microdosing comparison group adds substantially to a growing body of literature attesting to putative salutary effects of microdosing for mental health and mandate further research with more rigorous longitudinal designs such as randomized clinical trials and large cohort studies. Indeed, although the present study identified statistically significant differences in psychiatric symptom severity based on microdose status, these effects were in the range of effects typically characterized as *small*^35^. Any conclusions regarding the clinical import of these findings should consider these small effects and the limitations inherent in self-reported effects and cross-sectional design.

Microdosers and non-microdosers evinced interesting patterns of differences with regard to the use of other substances. Although both groups in our sample demonstrated rates of cannabis use and large-dose psychedelic use that exceeded what might be expected in a general community sample in Canada^36^, the USA^37,38^ and Europe^39,40^, microdosers were less likely to use alcohol regularly and were more likely to abstain from alcohol entirely. In light of the status of alcohol being among the most harmful psychoactive substances from both a personal and public health perspective^41^, the association between microdosing and low levels of alcohol use appears congruent with the broader health and wellness accentuating motivations for microdosing. Similarly, our finding that microdosers were more likely to abstain from the use of nicotine is also congruent with reducing harms associated with the use of psychoactive substances. Indeed, more than 25% of respondents endorsed the reduction of problematic substance use as a motive for microdosing.

In contrast to these lower rates of tobacco and alcohol use, microdosers were more likely to endorse frequent cannabis use. However, although frequent cannabis use may be associated with the development of cannabis related problems^42^ it is also a marker of therapeutic use^43,44^. As such, although the present study did not directly assess medical versus non-medical intentions of cannabis use, the prominence of therapeutic motives such as reducing anxiety and depression among microdosers suggests the possibility that frequent cannabis use may also reflect similar salutary intent. Future research that examines microdosing should more carefully examine the co-use of cannabis and microdosing and explicitly query therapeutic versus non therapeutic motivations for cannabis use. Similar considerations might also apply to the high levels of large-dose psychedelic use. Moreover, although we did control for prior psychedelic use broadly, further research examining the interaction between large-dose and microdose psychedelics, specifically considering factors such as large-dose frequency, dose and temporal precedence to microdosing, is warranted. The apparently imminent reintegration of large-dose psilocybin and other psychedelics into mainstream medicine prognosticates increased interest in and adoption of microdosing with therapeutic intent, making the rigorous evaluation of risks, benefits, and best practices for combining large-dose and microdose psychedelics a research priority, and several studies of this nature appear to be underway or in development^6,45^.

The promotion of mindfulness was the most highly endorsed motivation for microdosing among respondents who did not report mental health conditions, which suggests that efforts to enhance psychological well-being are primary even among those who are not microdosing to address more pronounced psychological distress. Other prominent motives included facilitating learning and creativity, and promoting health behaviors. Previous studies suggest that microdosing may further some of these desired outcomes, including reductions in mind-wandering and increased mindfulness^14,46^. Indeed, despite the stigmatization and criminalization of psychedelic substance use^47^, motivations for microdosing appear to be overwhelmingly therapeutic or wellness-oriented^13^.

In contrast to previous cross-sectional studies of microdosers^3,9,15^, our study identified a substantially higher proportion of psilocybin use relative to use of LSD. This finding may be specific to our sample but may also reflect shifts in the popularity and destigmatization of psilocybin that has both motivated and been amplified by recent policy changes such as the decriminalization of psilocybin possession in several jurisdictions and the apparently imminent approval of psilocybin medicines for psychiatric use in several contexts across North America and Europe^48,49^. Future studies are required to determine the extent to which these findings are anomalous or represent a broader shift in microdosing practices toward psilocybin and away from LSD. Future studies might also probe the generalizability of our novel findings that psilocybin was associated with more stacking with admixtures, with intensive and frequent microdosing, and with a greater focus on therapeutic intentions such as decreasing anxiety and improving sleep. Furthermore, in light of these differences future studies should clearly distinguish between psilocybin and LSD microdosing to avoid obscuring potentially important differences across substances.

Popular use of microdosing to address mental health concerns and enhance well-being has outpaced research on the risks and benefits of such use thereby mandating further research. Evidence derived from the use of larger doses of psychedelics suggests that psychedelics with predominantly serotonergic effects are safe when administered in controlled settings^50^. Preliminary results suggest that microdose practices have a similar safety profile to large-dose psychedelic use^6^. Nonetheless, the repeated use over long periods presents potential safety concerns distinct to microdose practices. For example, a potential adverse event specific to psilocybin microdosing are cardiac valvulopathies associated with the repeated activation of serotonin 5-HT_2b_ receptors via psilocin. Several medications, such as the diet medication Phen/Fen, have been restricted for similar concerns^51^ and although pre-clinical research have not suggested psilocybin related valvulopathy, this potential adverse effect requires consideration^8^. More broadly, further research is required to more confidently extrapolate safety data from infrequent use of large doses of psychedelics to the more consistent use of microdoses.

In general, our findings highlight the diversity of practices gathered under the umbrella of microdosing. Attempts to provide a comprehensive evaluation of the effects of microdosing need not only account for differences in substance, dose, frequency but should also consider the potential synergies implied by the widespread adoption of the practice of supplementing—or stacking—psychedelics with ingredients such as niacin and Lion’s Mane mushrooms. Indeed, although the present examination provides the most detailed account to date of the practice of stacking, our conclusions are nonetheless limited by a need for more fine grained detail regarding stacking practices. For example, animal models suggest that the impact of Lion’s Mane on brain functioning appears to be dependent on whether mycelium or fruitbody are consumed, such that mycelium promotes brain functioning whereas the fruitbody may have the reverse effect^52^; however our data did not permit this potential important distinction. As such, disambiguating the form of Lion’s Mane consumed by participants is an important distinction for future studies in order to minimize potentially contradictory effects. Further, the literature on stacking substances independent of psychedelic substances is itself limited, particularly with respect to clinical trials with human subjects^53,54^. In light of the limitations inherent in generalizing from animal to human models, proposals regarding the mechanisms underlying stacking remain speculative and warrant cautious interpretation. Thus, a promising avenue for future microdosing studies would be to distinguish the independent effects and synergies of psychedelic and stacked substances. Finally, although we identified differences in dose and frequency across psilocybin and LSD, interpretation of these apparent differences is limited by the lack of a consistent parameters for what constitutes low, medium, and high dosages of each respective substance. Moreover, interpreting apparent differences in frequency of use may be complicated by duration of effects.

The present study has several other important limitations including response bias related to participant self-selection, and recruitment through venues that are favorable toward psychedelic use, which may have resulted in overrepresentation in our sample by individuals who respond favorably to microdosing. Additionally, unavailability of an Android OS version of the QC application at the time of study limited participation to those with access to Apple devices. Given this potential bias, our characterization of the therapeutic use of microdosing should be interpreted with caution pending replication from research that employs a more systematic recruitment approach. Research that employs a more comprehensive psychodiagnostic approach would also increase our confidence of the generalizability of the findings to clinical populations who may consider microdosing to treat mental health concerns. Moreover, the present study did not assess microdosing practices engaged in prior to study completion. As such, we were limited in our ability to speak to the potential influence of more long standing microdosing practices among current microdosers and those with a history of microdosing. These limitations are counterbalanced by several strengths, including a substantially larger sample of microdosers than has been examined by prior research and that allowed for the more granular examination of relationships within and between distinct subgroups of microdosers. The inclusion of a large and comparable group of non-microdosers for the purpose of comparison is another strength and allowed a detailed examination of the associations between microdosing and mental health. More generally, these results highlight the potential and feasibility of studying microdosing and other potentially invisible or difficult to track substance use behaviors using a bespoke, mobile application which allows for the anonymous participation, self-enrolment, and the completion of assessments over time.

## Conclusion

This examination of a large international sample of adults highlights the prominence of therapeutic and wellness motivations for microdosing psychedelic drugs and identified lower levels of anxiety and depression among microdosers relative to controls. We also identified a diversity of microdosing practices with substantial variations in dose, frequency and use of combinations of psychedelic and non-psychedelic substances (i.e., stacking). Future research is warranted to better determine the impact of these distinct practices—and of microdosing more broadly—on the aspects of cognition, mood, and well-being which microdosing is intended to enhance.

## Data Availability

All data generated during and/or analyzed during the current study are available from the corresponding author on reasonable request.
